# Resectable Colorectal Cancer: Current Perceptions on the Correlation of Recurrence Risk, Microbiota and Detection of Genetic Mutations in Liquid Biopsies

**DOI:** 10.3390/cancers13143522

**Published:** 2021-07-14

**Authors:** Andreas Koulouris, Christos Tsagkaris, Ippokratis Messaritakis, Nikolaos Gouvas, Maria Sfakianaki, Maria Trypaki, Vasiliki Spyrou, Manousos Christodoulakis, Elias Athanasakis, Evangelos Xynos, Maria Tzardi, Dimitrios Mavroudis, John Souglakos

**Affiliations:** 1 Laboratory of Translational Oncology, Medical School, University of Crete, 70013 Heraklion, Greece; andreas.koulouris@gmail.com (A.K.); mimasf19@gmail.com (M.S.); tr.maria@gmail.com (M.T.); mavroudis@uoc.gr (D.M.); johnsougl@gmail.com (J.S.); 2 Department of Medical Oncology, University Hospital of Heraklion, 71110 Heraklion, Greece; 3 Faculty of Medicine, University of Crete, 70013 Heraklion, Greece; chriss20x@gmail.com; 4 Medical School, University of Cyprus, Nicosia 20537, Cyprus; nikos.gouvas@gmail.com; 5 Department of Radiation Oncology, Hygeia Hospital, 15123 Athens, Greece; vasiliki.spyrou21@gmail.com; 6 Department of General Surgery, Venizeleio General Hospital, Leoforos Knossou 44, 71409 Heraklion, Greece; christom7@gmail.com; 7 Department of Surgery, University General Hospital of Heraklion, 71110 Heraklion, Greece; eliasathanasakis@yahoo.gr; 8 Department of Surgery, Creta Interclinic Hospital of Heraklion, 71305 Heraklion, Greece; exynos@gmail.com; 9 Laboratory of Pathology, University General Hospital of Heraklion, 70013 Heraklion, Greece; tzardi@med.uoc.gr

**Keywords:** colorectal cancer (CRC), microbiota, circulating tumor cells (CTCs), cell-free DNA (cfDNA), recurrence risk, liquid biopsy, genetic mutations

## Abstract

**Simple Summary:**

CRC recurrence remains a great barrier in the disease management. Metastatic disease is a highly lethal malignancy. Novel biomarkers are urgently needed to address disease recurrence since specific genetic signatures can identify a higher or lower recurrence risk, thus serving as biomarkers and treatment targets. To a large extent, CRC is mediated by the immune and inflammatory interplay of microbiota, through intestinal dysbiosis. Clarification of these mechanisms will yield new opportunities, leading to appropriate stratification policies, and to more precise, personalized monitoring and treatment navigation. Under this perspective, early detection of post-operative CRC recurrence is of utmost importance. Ongoing trials, focusing on CTCs and, even more on ctDNA, seem to pave the way to a promising, minimally invasive, and life-saving monitoring, supporting personalized treatment and favoring patients’ quality of life.

**Abstract:**

Metastatic colorectal cancer (mCRC) remains a highly lethal malignancy, although considerable progress has resulted from molecular alterations in guiding optimal use of available treatments. CRC recurrence remains a great barrier in the disease management. Hence, the spotlight turns to newly mapped fields concerning recurrence risk factors in patients with resectable CRC with a focus on genetic mutations, microbiota remodeling and liquid biopsies. There is an urgent need for novel biomarkers to address disease recurrence since specific genetic signatures can identify a higher or lower recurrence risk (RR) and, thus, be used both as biomarkers and treatment targets. To a large extent, CRC is mediated by the immune and inflammatory interplay of microbiota, through intestinal dysbiosis. Clarification of these mechanisms will yield new opportunities, leading not only to the appropriate stratification policies, but also to more precise, personalized monitoring and treatment navigation. Under this perspective, early detection of post-operative CRC recurrence is of utmost importance. Ongoing trials, focusing on circulating tumor cells (CTCs) and, even more, circulating tumor DNA (ctDNA), seem to pave the way to a promising, minimally invasive but accurate and life-saving monitoring, not only supporting personalized treatment but favoring patients’ quality of life, as well.

## 1. Introduction

Colorectal cancer (CRC) recurrence is a major concern, whose likelihood appears to increase proportionally to the disease grading on diagnosis. Overall, 4–33% of patients undergoing CRC surgical resection will eventually relapse [1]. Particularly, in a recent population study in the US, the recurrence rate ranged from 5 to 6% in stage II colon cancer, 9.4–10.5% in stage II rectal cancer, and 14.6–17.7% in stage III CRC [2]. CRC recurrence usually occurs 2–3 years after initial treatment and pertains to locoregional recurrence (18%) in the pelvis or the peritoneum or distant metastases (78%), predominantly in the liver or lungs [3]. Several clinical and pathological factors offer a potential prognostic or predictive value, after colectomy. All these factors have been investigated in retrospective studies of population cohorts or posthoc subgroup analyses of randomized trials. Notably, the quality of surgery was not assessed in any of these studies, although it has been thoroughly documented that the complete mesocolic excision (CME) is of paramount importance.

A recent umbrella meta-analysis concluded that, out of 17 risk factors of locoregional recurrence, vascular invasion for lymph node metastasis (LNM) in pT1 CRC was the most evidence-based factor. Simultaneously, three factors were estimated to change the odds of the outcome at least 3-fold at a statistically significant level. These include tumor budding for overall recurrence in CRC, perineural invasion (PNI) for local recurrence in rectal cancer and MRI-detected extramural vascular invasion (mrEMVI) for distant metastatic recurrence in rectal cancer [4]. 

Gender and racial disparities have been observed in several aspects of CRC including recurrence. Proximal (right-sided) CRC, which is a more aggressive form of CRC with a higher relapse rate than distal (left-sided) CRC, is more common in women. A higher rate of recurrence among females has been also associated with the reproductive toxicity of anticancer drugs. This stated, temporary or permanent cessation of treatment can lead to relapse in female patients [5]. Hispanics, who have a higher incidence of carcinoid CRC, and African Americans tend to have increased CRC relapse rates compared to Caucasians, whose survival probability over five years is higher [6]. The incidence of CRC, and hence its recurrence, keeps increasing among Hispanic populations over the age of 50, while a decline is observed in all the other racial groups, in particular, from 60 years onwards [7]. Certainly, gender and racial disparities in CRC recurrence can partially be connected to modifiable risk factors, such as social status, migration, knowledge of family history and paucity of clinical data [8].

## 2. Genetic Mutations in CRC 

Genetic mutations implicated in the pathogenesis of CRC fall under two well-described hereditary forms: polyposis and non-polyposis CRC. Pathogenic variants of the adenomatous polyposis (APC) and the *MUTYH* gene belong to the polyposis form whereas, germline pathogenic mutations in DNA *MMR* genes (*MLH1*, *MSH2*, *MSH6*, *PMS2*) and *EPCAM* belong to the non-polyposis form. Additional associated genes include *POLE*, *POLD1* (oligopolyposis syndrome), *NTHL1*, *BMPR1A* and *SMAD4* (juvenile polyposis syndrome), *PTEN* (Cowden syndrome), and *STK11* (Peutz-Jeghers syndrome) [9]. Fusions of the genes encoding R-Spondin2 or R-Spondin3 have been observed in up to 10% of CRC [10]. On top of the involvement of genetics in CRC pathogenesis, there is an increasing interest in the mutations contributing to its recurrence. 

### 2.1. Genetic Mutations Associated with CRC Recurrence

Currently, the major biomarkers used in CRC clinical management are *KRAS*/*NRAS*, and *BRAF* oncogene mutations which predict resistance to anti-*EGFR* therapy in metastatic CRC (mCRC). The microsatellite instability (MSI) status is also used as a prognostic marker in stage II disease and as a predictive marker for the effectiveness of anti-PDL1/PD1 antibodies in mCRC [11,12,13]. Intending to address CRC recurrence, new and highly sensitive as well as specific, cost-effective and minimally invasive genetic biomarkers are needed. Such an approach could rescue patients with a good prognosis from the administration of toxic chemotherapy and lower the cost of management in early (stage II–III) CRC patients, by both decreasing the management cost for the side effects and intensifying the treatment approach to those at high recurrence risk. The available evidence has focused either on mutations associated with lower recurrence rates and better outcomes or on mutations associated with more frequent recurrence and decreased overall survival (OS) (Table 1). 

### 2.2. Mutations Associated with a Lower CRC Recurrence Rate

Studies focusing on CRC adenocarcinomas have detected a recurrent *VTIA-TCF7L2 fusion* in nearly 3% of cases [14]. Interestingly, recurrently mutated genes have been associated with longer survival. Next-generation sequencing (NGS) studies in CRC associated *CDH10*, *COL6A3*, *SMAD4*, *TMEM132D*, *VCAN*, *FAT4* and *DOCK2* polymorphisms with lower recurrence rates and up to 80 months of OS extent, whereas patients lacking these polymorphisms tended to survive for up to 40 months [15]. Kim et al. investigated 1160 genes in samples from 130 patients with CRC in an effort to predict systemic recurrence. This analysis yielded 11 genes, *AK2*, *CDC25A*, *HSPB1*, *BID*, *EIF4A2*, *ITGB1*, *MAP4K4*, *MMP12*, *RHOC*, *PTGES3* and *TERF2IP*, as the genetic signature associated with the longest disease recurrence-free survival (RFS). The prognostic potential was lower in patients with stage II–III CRC, which suggested higher effectiveness for genetic testing early in the disease [16].

### 2.3. Mutations Associated with a Higher CRC Recurrence Rate

*PIK3CA* mutations were associated with disease recurrence and poor survival in stage II–III colon cancer [17]. Disease recurrence has also been correlated with *LGR5* gene mutations in stage II CRC patients following curative surgery [18]. Aspirin and NSAIDs have been shown to reduce recurrence rates in patients with *PIK3CA* mutations [19]. A prospective study by Lan et al., analyzing samples from 1227 stage I–III CRC patients post-resection, indicated that *APC*, *BRAF* and *NRAS* mutations were present in patients with earlier recurrence and worse outcomes [20]. Vakiani et al. analyzed the genetic signature of distant metastases and anastomotic recurrence in 14 patients. They reported a total of 254 mutations, including 138 in MSI-stable disease, with *APC*, *KRAS*, *TP53*, *PIK3CA*, *ATM* and *PIK3R1* being the most common. There were significant differences between the genetic underpinnings of distant and locoregional recurrence-associated mutations [21]. Schweiger et al. reported that *KRAS* mutations were associated with a higher risk of lung recurrence in patients who underwent R0 CRC resection. This study failed to associate this data with *EGFR* mutations, but the authors have suggested this combination as a promising hypothesis for future and larger studies [22].

A body of research has paid attention to distant recurrence in the form of colorectal-liver metastasis (CRLM). *KRAS* mutations have been identified as an independent prognostic factor of recurrence in synchronous CRLM [23]. Moreover, Brunsell et al. demonstrated that *KRAS* mutations have been associated with poor survival following partial liver resection in 108 patients. The same was demonstrated in the case of combined *KRAS/NRAS/BRAF* mutations [24]. Mateo et al. enrolled 93 patients and indicated the complete or hybrid loss of mismatch repair expression as a risk factor for intrahepatic recurrence of CRLM, post-hepatic resection [25].

Novel research focuses on the use of genetic mutations, not only as biomarkers but also as treatment targets [26]. Attention is also paid to epigenetics. The gradual accumulation of epigenetic alterations in the physiological intestinal epithelium has been shown to contribute to the initiation and promotion of CRC. Emerging evidence has pointed to the gut microbiota and its involvement in tumorigenesis and metastasis through DNA methylation, histone modifications, and non-coding RNAs [27]. A recent study has illustrated the abundance of microorganisms associated with CRC, such as *Fusobacterium*, *Bacteroides*, *Parvimonas*, and *Prevotella* spp. in CRC specimens with *KRAS* mutations or MSI status [28]. Hence, evidence regarding the involvement of gut microbiota in CRC recurrence is complementary to the existing knowledge about the genetics of CRC recurrence.

### 2.4. The Role of Cancer Stem Cells (CSCs) in CRC Recurrence

CSCs consist of a population of cells that have the potential to foreshadow CRC recurrence and metastatogenesis via resistance to chemotherapy and immune evasion. A plenitude of emerging biomarkers may play a crucial role in the formation of CSCs colonies regarding CRC, such as prominin-1 (CD133), CD44 antigen (CD44) and specific micro ribonucleic acids (miRNAs) [29]. Prognostic CRC stem cell markers can be categorized into three major groups: a. the surface markers, such as CD44, CD133 and CD166, b. the surface markers, for instance, the Leucine-rich repeat-containing G protein-coupled receptor 5 (LGR5) and c. the intracellular markers, like ALDH, Achaete-Scute Homologue 2 (ASCL2), Nanog, Oct-3/4, Sall4 and Sox2 [30]. Particularly, the prognostic marker CD133 seems to contribute to the resistance to chemotherapy in CRC CSCs. Its combination with other markers, such as the CD44, has been documented to increase its reliability [29]. Furthermore, the biomarkers CD166, CD44, and LGR5 have been noticed to be overexpressed in stage III and metastatic CRC. More specifically, it is worthy of further notice that the elevated expression of CD166 and ASCL2 gene seems to be related to high recurrence risk even regarding stage I and II CRC [31].

As far as miRNA biomarkers are concerned, a striking example is the miR-486-5p, whose levels have been revealed to be downregulated in patients with metastatic CRC. According to novel genomic data, the potential inhibitory role of this emerging diagnostic biomarker could be attributed to the negative regulation of the expression of stemness-related transcription factors and the major pathways of CSCs: TGF-β, Notch, Hedgehog, and Wnt [32].

## 3. Microbiota in CRC

The understanding of gut microbiota and its role in human health and disease has advanced in the last decades. The ideal symbiotic interplay of trillions of bacteria, viruses and eukaryotes inhabiting the gastrointestinal tract can be disrupted by intrinsic and extrinsic factors, such as immunity, nutrition, general life style and medication, triggering a plethora of pathogenetic cascades [33]. In patients with CRC, sequencing studies have reported changes in the composition and ecology of gut microbiota. Animal models of CRC have yielded the involvement of microbial compositional and ecological changes in CRC. Other studies have stressed the roles of particular bacteria in CRC carcinogenesis, such as *Fusobacterium* *nucleatum* sp. and certain strains of *Escherichia* *coli* and *Bacteroides* *fragilis* spp. [34]. Other studies have suggested *Streptococcus bovis*, *Enterococcus faecalis* and *Peptostreptococcus* *anaerobius* as candidate pathogens for CRC development [35]. Conditions characterized by microbial dysbiosis, such as Crohn’s disease, have also been associated with a higher risk of CRC development [36]. At a mechanistic level, it seems that microbial dysbiosis can increase the secretion of inflammatory mediators such as tumor necrosis factor, nuclear factor kappa B, interleukins, and interferons, leading to mutations and dysplasia [37]. Even when tumorigenesis is not linked to intestinal flora, particular bacteria may interact with the tumor via oncometabolites (e.g., l-2-hydroxyglutarate, succinate, fumarate) which enhance cancer progression [38]. The latter is a double-edged sword, given that other bacterial metabolites such as acetate, butyrate, and propionate have been shown to downsize tumor growth [39]. On these grounds, the microbiome is now regarded as an additional CRC tool regarding the armamentarium of potential biomarkers [40], with guaiac-based fecal occult blood tests (gFOBTs), fecal immuno-chemical tests (FITs) and multitarget stool DNA (sDNA) testing being integrated into state of the art diagnostic testing protocols [41]. Therefore, conceptualizing the microbial components of recurrence may have the potential to improve disease monitoring. 

### The Role of Gut Microbiota in CRC Recurrence

Mounting evidence focuses on the contribution of gut microbiota in CRC recurrence. Various factors, from surgical resection to diet, general lifestyle or medications have been shown to alter the microbial population of the intestines, promoting dysbiosis, subsequent inflammation and tissue damage. Many studies have focused on separate microorganisms, while others have pointed out the need to address CRC recurrence in the context of microbial communities’ interaction. Going deeper than the general statements on dysbiosis and inflammation, specific pathogens have been associated with oncogenic processes [42]. In particular, *E. coli* sp. can initiate oncogenic DNA damage by producing colibactin, a secondary genotoxic metabolite [43]. The production of the oncogenic colibactin has been associated with bacterial polyketide synthetase (*pks*) in both organoids and human studies [44,45]. The identification of *pks+ E. coli* sp. can be further assessed as a potential biomarker indicating the need for either more intense follow-up or aggressive treatment in the first place. *B. fragilis* sp. contributes to tumorigenesis and recurrence via enterotoxin-induced cell proliferation and oncogenic inflammation [46]. *F. nucleatum* sp. promotes CRC progression and relapse by means of Fap2 and FadA adhesins. Both are involved in cancer cells proliferation, antitumor immune evasion and metastasis [47]. Regarding the potential mechanism of recurrence, it is well established that, following surgical resection, cancer cells exfoliate in the intestinal lumen and set the fundaments of recurrence [48]. Simultaneously, the resection per se affects the composition of the microbial flora for at least three months. A recent clinical study reported an increase in *Abacteroides*, *Streptococcus*, and *Ruminococcus* spp., following endoscopic resection of adenomas [49]. In such a microenvironment, the ability of exfoliated cancer cells to proliferate and form new tumors is linked to the microbial flora. Bacteria with collagenase activity, particularly *Enterococcus faecalis*, *Pseudomonas aeruginosa* and *Serratia marcescens* spp., can tolerate or even promote their differentiation into more aggressive phenotypes [50,51,52]. Although the mechanistic underpinnings of bacteria-assisted recurrence are yet to be established for most bacteria, a recent meta-analysis validated the association of worse prognosis and recurrence after surgery with abundant populations of *F. nucleatum* and *B*. *fragilis* spp. [53]. 

In vitro and in vivo studies have focused on *F. nucleatum* as a potential promoter of CRC recurrence. At a mechanistic level, it has been shown that *F.* *nucleatum* sp. contributes to the epithelial–mesenchymal transition (EMT) by binding to E-cadherin expressed on adenocarcinomas, promoting its internalization into the cytoplasm, activating the β-catenin complex and triggering a cascade of inflammatory genes [54]. The persistence of *F. nucleatum* sp. in patients with rectal carcinoma is associated with high locoregional relapse rates, potentially due to immunosuppression in 143 patients [47]. The carcinogenic effect of *F*. *nucleatum* in CRC recurrence seems to be enhanced by the presence of oral commensals like *Bacteroides*, *Salmonella*, and *Prevotella* spp., although the capacity of these bacteria to enhance tumor progression themselves in CRC tissue is debatable [55,56]. On top of these, *F. nucleatum* has been shown to induce resistance to recurrent CRC treatment with oxaliplatin and/or 5-Fluorouracil (5FU), decreasing the five-year survival rate of these patients by less than 10% [57]. Resistance is mediated by activation of the *BRIC3* gene, following TLR4/NF-κB mediated infection of CRC cells by *F. nucleatum* [58].

Indirect evidence on the role of gut microbiota in CRC recurrence derives from nutrition and administration of medicinal regimens, such as antibiotics and colostrum preparations. A preclinical study in mice suggested that rich in fat Western diet-induced proliferation of *E. faecalis* and *Proteus mirabilis* spp. contribute to tumorigenesis post-CRC resection. Moreover, consumption of red meat has been linked to an increase of *F. nucleatum* sp. promoting CRC tumorigenesis through the activation of oncogenes and inflammatory mediators. The study was initially performed on Chinese subjects and later validated with tissue specimens from Europe [59]. A course of antibiotics to reduce the population of these bacteria failed to reduce the development of recurrences and enhanced the colonization of the gut with *Candida parapsilosis* sp., a potentially tumorigenic fungus. Eventually, administration of Pi-PEG reduced tumor formation and promoted symbiosis in the colon microbiome [60]. An additional study on mice receiving high calorie Western diet (high fat, no fiber, and decreased minerals and vitamins), reported an astonishingly high (88%) level of recurrent CRCs following surgical excision of the primary tumor [60]. KMP01D, a colostrum preparation, demonstrated beneficial ex vivo effects on inflammatory cytokine responses in patient-derived blood mononuclear cells and contributed to the apoptosis of immune cells collected from CRC patients. KMP01D appears as a promising treatment strategy in regulating stage-dependent local and systemic inflammation associated with gut microbiota dysbiosis in CRC patients [61]. This finding is in accordance with a body of evidence suggesting the contribution of inflammatory cytokines in recurrence and the potential of such preparations to mitigate this [62,63]. The role of antidiabetic medications in CRC is also of high importance, given the high prevalence of type 2 diabetes mellitus worldwide, and particularly among CRC patients. A recent study has proposed the so-called metformin–gut microbiota–CRC axis concept based on evidence suggesting that diabetic patients receiving metformin had a lower rate of CRC diagnosis or recurrence [64]. Studies on healthy individuals have established that metformin affects the gut microflora reducing the populations of *Intestinibacter* and *Clostridium* spp. and contributing to the increase of *Escherichia*, *Shigella* and *Bilophila* *wadsworthia* spp. within one year of administration [65]. In this context, a large-scale population study on 6650 patients from the US showed that metformin administration was associated with decreased CRC odds—mainly in rectal cancer—among diabetic individuals [66]. Further research in this field can elucidate the mechanistic–microbiological underpinnings of the effects of metformin on CRC.

Interventions to modulate the microbial flora in patients who underwent CRC surgical resection need to be further investigated based on these findings. It is important to harmonize these interventions with the available evidence on the taxonomy of microbial species, which are more abundant in specific types of CRC. These include bacteria of *Prevotella*, *Eubacterium*, *Dorea*, *Fusicatenibacter*, *Howardella*, *Butyricicoccus*, *Anaerococcus*, *Alloprevotella*, *Faecalibacterium*, *Roseburia*, and *Sutterella* genera. *Granulicatella*, *Burkholderiales*, *Flavonifractor*, *Coprobacillus*, *Parabacteroides*, *Anaerotruncus*, *Akkermansia*, *Allisonella*, and *Alistipes* genera are more abundant in the sigmoid colon than in rectal cancer [67]. Remarkably, it has been reported since 2014 that invasive multi-bacterial biofilms were detected in 89% of right-sided CRC (RCC) but in only 12% of left-sided CRC (LCC) and the former were related to aggravated crypt epithelial cell proliferation in normal colon mucosa, diminished intestine epithelial cell line E-cadherin, increased epithelial permeability, and enhanced activation of IL-6 and STAT3 [68]. This model reflects the relationship between gut microbiota and CRC, given that biofilms seem to promote the precancerous inflammation of the tissue [35]. There are a plethora of molecular differences between RCC and LCC besides the more frequent formulation of biofilms in RCC. More specifically, *Prevotella*, *Selenomonas* and *Peptostreptococcus* spp. are more abundant in RCC patients. On the other hand, *Fusobacterium*, *Escherichia/Shigella*, and *Leptotrichia* spp. are more abundant in LCC. Moreover, CpG island methylator phenotype-high and MSI-high CRC are more likely to occur in RCC. The mutation rates of oncogenes and tumor suppressor genes also differ between RCC and LCC patients. Consequently, the intestinal microbiota seems to be another factor that emphasizes on the different origins and nature between RCC and LCC [69,70]. 

**Figure 1:**
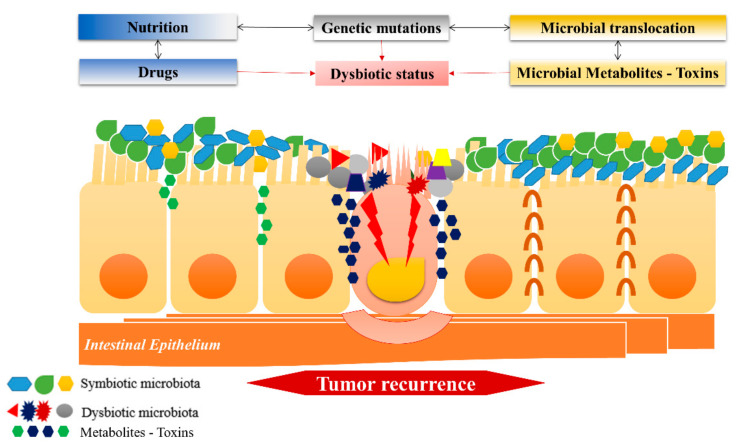
External factors (nutrition, medications), as well as intrinsic factors (microbial translocation and metabolites), affect commensal microorganisms (*F. prausnitzii*, *Bifidobacteri*) enabling bacteria associated with a higher risk of tumor recurrence (*F. nucleatus*, *E. faecalis*, *P. mirabilis*, *E. Coli*, *Abacteroides*, *Salmonella* etc.) to prevail and modulate the intestinal microenvironment in a plenitude of ways promoting neoplastic cellular growth.

As far as microbiota interventions are concerned, they can entail specialized nutrition, treatment rounds with antibiotics and even fecal transplantation, as long as the post-operative condition of the patients allows so [71]. The administration of ursodeoxycholic acid (UDCA) is a promising option for male patients. A recent study, based on the daily administration of UDCA in patients with colorectal adenomas, reported that an increase in the presence of *F. prausnitzii* sp. led to a reduction of *Ruminococcus gnavus* sp. decreasing the relapse risk of adenomas [72]. This effect and the microorganisms implicated are worth studying in CRC. It is also noteworthy that the use of antibiotics to modulate microbiota and decrease the likelihood of recurrence has backfired in a clinical study, where penicillin was associated with a higher risk of esophageal, gastric and pancreatic cancer whereas, sulphonamides and tetracycles were associated with prostate and breast cancer [73]. Iron supplementation, a therapy frequently prescribed post-CRC resections is a point of inquiry as well. A recent study showed the supremacy of *intravenous* over *per o*s iron supplementation in mitigating inflammation around the tumor site by modulating the local microbial environment. The study included samples from 40 individuals, hence validation in larger cohorts is needed [74]. Nonetheless, the aforementioned studies indicate that even frequently prescribed medications such as antibiotics or iron supplementation need to be reconsidered in the context of microbiota-mediated CRC recurrence (Figure 1).

Several ongoing trials focus on nutritional and other interventions to evaluate whether intestinal microbiome is related to CRC recurrence and whether microbiome alterations can somehow influence CRC. Particularly, the Be Gone trial is an active study that enrolled obese survivors with a history of cured CRC or a colorectal polyp for the evaluation of whether eating canned, precooked beans can improve the levels of beneficial bacteria in the gastrointestinal system and reduce the effects of obesity on cancer risk, recurrence and survival [75]. The FAMiLI trial has also enrolled patients with CRC aiming to investigate the differences in human microbiota between subjects with and without colorectal adenoma or CRC [76]. Another study recruits patients with metastatic cancer who receive chemotherapy or immunotherapy in order to compare the changes in gut microbiome composition and in the metabolic activities with response to chemotherapy or immunotherapy, as well as their toxicity [77]. Furthermore, the Metabiomics Colon Cancer Clinical Research Study examines the relation of the gut microbiome to colonic neoplasia, whereas another group focuses on the metagenomic assessment of the gut microbiome taking into account dietary factors regarding patients with Lynch syndrome and other hereditary colonic polyposis syndromes [78,79]. Finally, the core of the prospective study of our group, Cologramme, consists of an extensive research database, which will be used for the assessment of the correlations between detectable genetic alterations and the microbiota in plasma and stool specimens of patients with localized (stage II–III) CRC aiming to explore their impact on CRC recurrence risk and survival.

## 4. Liquid Biopsies: Circulating Tumor Cells and Cell-Free DNA in CRC

Although tumor biopsies is the gold standard for the diagnosis of CRC, liquid biopsy assessing the presence of CTCs or circulating tumor DNA (ctDNA) in the patient’s serum provide many chances for early diagnosis of the disease and its potential recurrence [80,81,82]. 

CTCs stem from primary tumors and are released in the peripheral blood during CRC progression. Even a single CTC can lead to distant metastasis. Hence, CTCs have a great potential in cancer diagnostics, staging and recurrence monitoring. CTCs can also serve as treatment targets to mitigate the spread of the disease. Their clinical applications are still limited. They have been studied in CRC treatment monitoring—while other studies have assessed their potential in the diagnosis of gastric, esophageal or pancreatic cancer [83]. The most recent study, including 56 CRC patients, suggested that mutations not detected in primary tumors can be identified in CTCs’ DNA, highlighting their potential in complementing gene analysis. The same study suggested that combination analysis with ctDNA improves sensitivity, although the detection of cancer-specific mutations was superior in cell-free DNA (cfDNA) in comparison to CTCs’ DNA [84].

ctDNA is a fraction of the cfDNA, fragmented DNA detected among the non-cellular blood components of healthy individuals. Among patients with cancer, ctDNA is released in the bloodstream by tumor cells in 150~200 base pair fragments, comprising a small portion of the total cfDNA. The significance of ctDNA lies in its ability to maintain epigenetic traits and carry tumor-specific mutations, which are detectable in peripheral blood samples [85,86]. Relevant clinical applications for the early-stage disease include the detection of ctDNA methylation and the assessment of circulating protein levels and mutations in cfDNA or the analysis of fragment length distribution of ctDNA types. Droplet digital polymerase chain reaction (ddPCR) and NGS are used for recurrence diagnosis. Amplicon-based deep sequencing and ctDNA quantification are used to monitor treatment response and ddPCR, NGS and ctDNA mutations serve as indicators of the therapeutic resistance in metastatic patients [86,87,88,89].

Both CTCs and ctDNA consist of liquid biopsies. Soon, their significance is expected to rise because they combine efficacy and safety. Liquid biopsies are capable of detecting CRC-associated mutations, indicating the tumor burden and assessing the heterogeneity of the tumor. In contrast to tumor biopsies, liquid biopsies are non-invasive and non-traumatizing. They are obtained from serum or urine samples; therefore, they pose minimal risk to the patient, their sampling procedures do not require highly specialized personnel and they are time- and cost-saving [90].

Despite these advantages, liquid biopsies are subject to limitations. More evidence from large-scale cohort studies is required to establish their efficacy in CRC, at subtype and stage level. To date, the clinical use and efficacy of liquid biopsy techniques is limited, as shown by a joint assessment of the American Society of Clinical Oncologists and the American College of Pathologists, and this poses additional challenges to their validation in clinical settings [91]. Research funding and insurance coverage of liquid biopsies in contemporary healthcare systems constitute additional challenges. Their early integration in clinical practice encompasses the risk of missing diagnoses or falsely alarming clinicians and patients leading to burdenful and risky aggressive treatment approaches. The latter consists of an additional ethical limitation to their use [92]. The available evidence about the efficacy of both types of liquid biopsies in CRC (CTCs, ctDNA) is presented in the following subsections.

### 4.1. Association between Liquid Biopsy and CRC Recurrence

Proof-of-concept studies indicated that circulating cfDNA levels have a greater dynamic range and greater correlation with changes in tumor burden than CTCs [93]. In this section, evidence regarding both ctDNA and CTCs is presented.

#### 4.1.1. Circulating Tumor DNA (ctDNA)

A consensus paper from the Gastrointestinal Cancer Therapy Expert Group has suggested the use of ctDNA for the detection of minimal residual disease in patients with early-stage CRC, following successful resection [94]. Such a consensus comes on top of a growing body of research, which has explored the potential of ctDNA in various contexts of resectable CRC. Nakamura et al. analyzed samples from 180 patients with resectable CRC in Japan. Their findings indicated that *KRAS* mutated ctDNA was independently associated with inferior recurrence-free interval (RFI) and recurrence-free survival (RFS) and suggested that pre-operative ctDNA detection needs to be taken into consideration to optimize post-surgical management [95]. Boysen et al. studied prospectively serum samples from 35 CRC patients two weeks after surgical resection. *BRAF*, *NRAS* and *KRAS* mutated ctDNA were associated with CRC recurrence within a median of 273 days [96]. Symonds et al. compared ctDNA and the carcinoembryonic antigen (CEA) 12 months after the recurrence of initially resected CRC in 144 patients. Their findings elucidated the supremacy of ctDNA over CEA both in terms of sensitivity and prediction of recurrence [97]. Benesova et al. compared the capacity of early recurrence detection among ctDNA, imaging and elevated tumor markers in 47 patients with detectable ctDNA at the time of CRC resection. ctDNA has been detected in all recurrent cases, whereas cases were missed by imaging and conventional tumor markers [98]. The latter supports the inclusion of ctDNA in clinical practice, as long as its supremacy over the existing follow-up means is validated in larger studies. 

Published and ongoing prospective population-based studies can increase the evidence about ctDNA in resectable CRC. Tie et al. conducted a multicenter, population-based cohort biomarker study in 96 stage III CRC patients in Australia; 21% of the post-surgical samples contained ctDNA and these had inferior RFS with 3 years of follow-up. The correlation between ctDNA detection and poor outcomes was independent of other clinicopathological factors yielding ctDNA analysis after surgery as a potential prognostic marker in stage III colon cancer [99]. Findings from a similar prospective study suggested that ctDNA detection in 125 stage I–III Danish CRC patients’ post-resection was linked to a 17-fold increased risk of relapse. It was noteworthy that in this population, relapse occurred up to 16 months earlier than standard-of-care imaging follow-up [100]. Larger studies are necessary to assess whether more aggressive follow-up of patients with detectable ctDNA could improve their outcomes. This might be achieved in a multitude of prospective randomized studies that have been listed in the CIRCULATE-Japan adaptive trial platform. More specifically, the GALAXY study monitors recurrence using ctDNA in 2500 patients with resectable stage II–IV CRC [101]. The VEGA randomized phase III trial investigates whether post-operative surgery alone is non-inferior to conventional chemotherapy in patients with high-risk stage II or low-risk stage III colon cancer, whose ctDNA status is negative 4 weeks after curative surgery [102]. Conversely, the ALTAIR double-blind, phase III trial focuses on patients with positive ctDNA assessing whether trifluridine/tipiracil is more effective than placebo, post-resection [103]. Simultaneously, the BESPOKE CRC prospective study enrolls stage II/III CRC patients, post-resection. Although CIRCULATE-Japan and BESPOKE focus on different populations, both will be based on the SIGNATERA^®^ ctDNA Test, which will make the comparison of the results easier [104]. In the same context, the MEDOCC-CrEATE trial of the Prospective Dutch ColoRectal Cancer cohort has recruited 1320 stage II CRC patients to assess the effectiveness of adjuvant chemotherapy based on the ctDNA status [105]. Since 2020, multiple studies and preliminary data suggest that ctDNA-guided risk stratification for adjuvant treatment seems to be more efficient than existing clinicopathologic prognostic indicators, it may also guide the therapeutic decision of dose escalation or de-escalation and, finally, regular ctDNA monitoring after the completion of definitive therapy can potentially lead to significantly earlier recurrence detection [106]. Finally, the promising study, Cologramme, investigates the correlations among the risk recurrence, the detection of genetic mutations, the microbiota and the CTCs and ctDNA in patients with resectable CRC. This could be beneficial to build highly relevant predictive and prognostic models in which CTCs and ctDNA variables will be included. 

#### 4.1.2. Circulating Tumor Cells (CTCs)

Yang et al. calculated the preoperative controlling nutritional status (CONUT) score and CTC status in a retrospective analysis of 160 CRC patients under curative resection [107]. Their analysis suggested that a high CONUT–CTC score was associated with poor prognosis and high recurrence risk in stage III but not in stage II CRC patients. Arazzubi et al. followed up 44 patients for 60 months and indicated that preoperative detection of ≥2 CTCs was associated with recurrence and poor prognosis despite curative resection [108]. Conversely, Yang et al. studied the outcomes of 211 stage I-III patients’ post-surgical resection and suggested that post-operative detection of CTCs was associated with poor prognosis [109]. Chang et al. compared a multi-Gene Biomarker Chip detecting CTCs and CEA levels in 298 stage I–III CRC patients after curative resection. Both markers were strongly associated with relapse, however, the specificity, the positive and negative predictive value and the accuracy of CTCs detection for post-operative recurrence were significantly higher [110]. Tseng et al. investigated CTCs in mesenteric circulation and around the tumor mass of 229 stage I–III CRC patients. They reported that CTCs in mesenteric circulation could be useful indicators of recurrence [111]. Galizia et al. investigated the presence of EpCAM/CD326 positive CTCs in 76 stage I–III CRC patients undergoing surgical resection. Their analysis yielded the potential of high levels of CTCs post-operatively to predict CRC recurrence. Again, post-operative CRC detection was superior to pre-operative detection [112]. In a study by Rahbari et al., CTCs were shown inferior to circulating angiogenic factors (CAF) for the prediction of recurrence following stage I–III CRC surgical resection in 107 patients [113]. Lu et al. used human telomerase reverse transcriptase, cytokeratin-19, cytokeratin-20, and *CEA*mRNA to detect CTCs in 141 patients with stage II–III colon cancer undergoing curative resection. CTCs were strongly correlated with early relapse [114]. Vardakis et al. demonstrated that peripheral blood *CEACAM5*mRNA-positive cells appear as an adverse prognostic factor associated with poor outcomes in patients with resectable CRC. The authors reported that the presence of these cells in serum samples of 256 patients with operable CRC was correlated with a higher CRC recurrence rate. The same study highlighted *CEACAM5*mRNA-positive cells as an independent prognostic factor for limited disease-free survival time [115]. A subsequent study from the same research group indicated an even higher correlation between *CEACAM5*mRNA-positive cells and decreased progression-free survival time in CRC patients with *KRAS* and *BRAF* mutated tumors [116]. Overall, it seems that researchers have paid more attention to cfDNA and less attention to CTCs, since 2018. Several ongoing clinical trials, especially from 2020 and onwards, are monitoring the ctDNA status regarding both recurrence and treatment choices. In anticipation of the endpoints of these trials to be met, ctDNA seems to claim the position of the cornerstone in monitoring resectable CRC in the near future [106,117]. However, the existing body of evidence regarding CTCs enabled researchers to investigate some innovative concepts. In particular, in 2018, Brown et al. studied the effect of various patterns of physical activity in 23 patients with ≥1 CTCs following stage I-III CRC resection. They concluded that exercise was associated with a decrease in CTCs within 6 months [118]. This hypothesis-generating study needs to be further investigated in the future but offers encouraging prospects in the intersection of preventive lifestyle modifications and molecular diagnostics. To sum up, liquid biopsy seems to have an emerging role in the management and the follow-up of resectable CRC (Table 2).

## 5. Cologramme Outline

The Cologramme project, is a prospective ongoing study, for the evaluation of all above-mentioned biomarkers, in patients that underwent a curative colectomy. The Cologramme is based on discrete research, development and innovation streams including: (1) sample collection, electronic case report form (eCRF), the electronic database formation; (2) identification of somatic and germline variants; (3) microbiome analysis in plasma, tissue and stools; (4) liquid biopsy based on CTCs and cfDNA in samples before and after surgery and at the end of adjuvant treatment; (5) computational analysis.

Cologramme has prospectively enrolled 100 patients with colon and 50 patients with rectal adenocarcinomas, stages II and III, in 24 months from four network centers of the Gastrointestinal Cancer Study Group (GIC-SG) [119]. All surgical specimens were accessed for the quality of surgical resection according to the international guidelines [120,121]. Only specimens scored as mesocolic or mesorectal plan were submitted for molecular analysis. This approach eliminates biases in risk assessment from inadequate surgical quality. Collection of stool specimens has to be performed pre-operatively and together with the corresponding tissue sample will be analyzed on an NGS platform. We will analyze 150 prospectively collected samples (stool and tissue), in comparison with a control group consisting of 15 patients with inflammatory bowel disease (stool and tissue), 15 with adenomas (stool and tissue), and 30 healthy donors (stools). Blood samples for CTCs and plasma was obtained prospectively, pre-operatively, 4–8 weeks after colectomy, and 6 months after colectomy. Extensive epidemiological and clinical data were collected and recorded in the electronic database. Those include place of residence, origin, race, education, job, age, weight, gender, smoking habits, physical activity (hours/week), routine diet and behavior, probiotics or other supplement consumption, water supply, cohabitation with animals, visits abroad in the last 12 months, eosinophils levels. In addition, detailed pathological findings will be included in the database: T status, number of retrieved and infiltrated lymph nodes, status of the apical lymph nodes, grade, subtypes, mucinous features, lymphocytic reaction, EMVI, tumor budding, distance of the tumor in mm for upper and lower edge, etc.

Formalin-fixed paraffin embedded (FFPE) tissue samples will be evaluated by a specialized pathologist and the cancer cells will be selected with the use of a piezoelectric micro-dissector. 

Assessment of single nucleotide variants, small deletions and gene fusions will be performed by using the commercialized Ion AmpliSeq Colon and Lung Cancer Research Panel v2 (Thermo Fisher Scientific, Wilmington, DE, USA). The panel includes primer pairs in a single pool for hotspots and targeted regions for 22 known genes (*KRAS*, *EGFR*, *BRAF*, *PIK3CA*, *AKT1*, *ERBB2*, *PTEN*, *NRAS*, *STK11*, *MAP2K1*, *ALK*, *DDR2*, *CTNNB1*, *MET*, *TP53*, *SMAD4*, *FBX7*, *FGFR3*, *NOTCH1*, *ERBB4*, *FGFR1*, and *FGFR2*) associated with colon and lung tumor tissue. Identification of microbiome will be done by 16S rRNA sequencing. For the 16S rRNA sequencing, primers directed against the 16S rRNA V3-V4 region will be designed to incorporate Ion Torrent-compatible sequencing adaptors. CTCs will be detected by RT-qPCR for *CEACAM5*mRNA according to a standard sensitive, repeatable and reproducible protocol established in our laboratory, both in the early and metastatic setting [115]. However, despite the proved prognostic value of CEACAM5, its epithelial origin does not allow mesenchymal cells or cells under epithelial-to-mesenchymal transition to be detected. The aim of the computational and statistical analysis is to exploit and to accent the Cologramme precision to calculation the risk of recurrence in CRC patients. Statistically, this list will contain a focused, targeted set of genetic alterations and their pathways that is likely to have the highest contribution to treatment resistance.

## 6. Discussion

As a consequence of increasing CRC prevalence due to aging of the population, increasing incidence and better survival rates, the incidence of new primary colorectal tumors will increase in parallel. In the past decades, significant progress has been made in understanding the molecular and genetic origin of CRC [122,123,124]. This major expansion of our knowledge has also contributed to the discovery of new therapeutic agents mostly in the metastatic setting. Although interesting associations have been made that might be of clinical relevance, the molecular classification of CRC is still in its early development [122]. To date, most available biomarkers are not recommended for use in the daily clinical practice. The only biomarker which is broadly used in clinical practice is *KRAS*/*NRAS*, and partly *BRAF*, oncogene mutations and the MSI status [125]. Such oncogene mutations predict resistance to anti-EGFR therapy in metastatic CRC, whereas MSI is used as a prognostic marker in stage II disease and as a predictive marker for the effectiveness of anti-PDL1/PD1 antibodies in the metastatic setting [126].

In order to cope with the increasing number of patients in the future, new and highly sensitive as well as specific, cost-effective and minimal invasive prognostic markers and molecular image techniques are warranted. New markers for stage II–III CRC patients are required in order to predict treatment outcome and to select patients that will be benefit for the administration of adjuvant treatment. Such an approach could rescue patients with good prognosis for the administration of toxic chemotherapy and lower the cost for the management of patients with early (stage II–III) CRC and decrease the cost of the management for the side effects. We anticipate that the results of such project will contribute to a further development of personalized treatment of CRC. 

**Figure 2:**
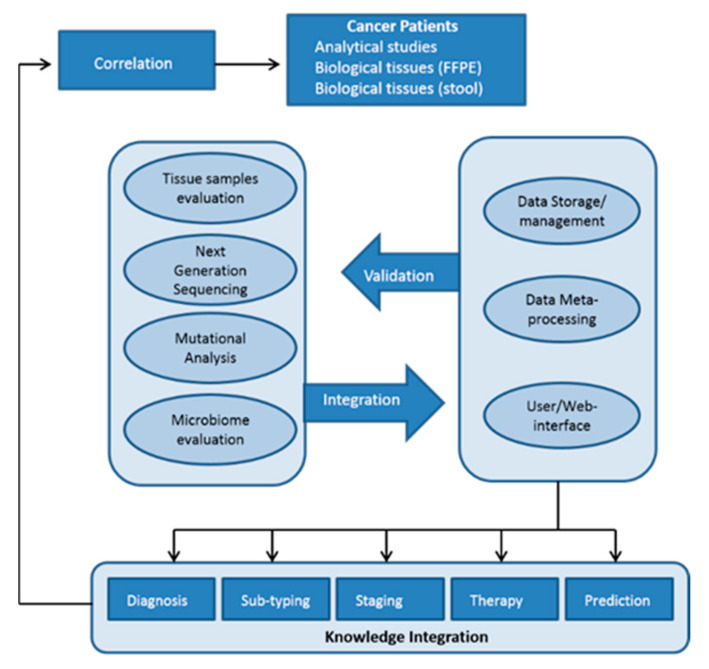
Graphical presentation of the tasks showing the way they inter-relate (Pert Chart).

The research databases that will be created in this project will contain a large variation of clinical data and stored biomaterial (blood, stool and tumor tissue) that will be used for the assessment of recurrence score. These markers may be used to (i) determine patient’s prognosis which could lead to more accurate diagnostic tests and efficient follow-up surveillance strategies and (ii) predict treatment outcome and guide the selection of the patients who will most likely take benefit from adjuvant treatment. It will give the opportunity to build highly relevant predictive and prognostic models for localized (stage II–III) CRC and explore their impact on CRC survival and the incidence of recurrence. Additionally, it could have a major impact on the design of more accurate and efficient follow-up surveillance strategies. Today, patients with early (stage II–III) CRC require at least a 6–7 years follow-up time on a regular basis, which subsequently leads to an increased use of health care. This burden may be reduced if patients can be categorized into those being at a low or a high risk of recurrence. Since good prognostic markers are lacking, all stage II–III CRC patients now undergo intensive follow-up including regular imaging and endoscopic assessment (Figure 2). 

As such, Cologramme is original at international level. It aims to provide a national and international accessible source of data on CRC. These data will be available for any research group or organization to perform scientific research, (internal) auditing and policy making. In order to guarantee that the data is only used for scientific, sociological and/or economical relevant research and is not performed by multiple parties simultaneously, the data from the project will only be accessible after permission of the research team of this project. The linkage between the longitudinal collected clinical data, the availability of biomaterials and the ability to perform basic research makes the current project a unique project that contains a large amount of data which will give the opportunity to conduct highly powered studies on translational research and overall to improve healthcare for all CRC patients. Finally, the results of Cologramme will be used for the rational design of confirmatory prospective clinical trial in which the recurrence score will be used as a stratification factor or as a toll for treatment (or no treatment) decisions.

In the future, exosome-derived miRNAs might be investigated and considered along with genetic mutations, microbiome and liquid biopsies. A growing body of evidence suggests that particular miRNA clusters (miR-17-92, miR-19a, miR-19b, miR-23a, miR-92a, miR-320a, and miR-4437) collected from blood, urine or even saliva samples can provide additional biomarkers of CRC recurrence risk stratification. In total, more than 20 miRNA clusters have been correlated with CRC recurrence in small-scale studies involving less than 100 patients by means of RNA sequencing (RNA-Seq) and/or qRT-PCR. However, the gold standard miRNA detection method and the sensitivity and specificity profile of the technique are yet to be determined [127].

## 7. Conclusions 

A new approach is proposed in the current work and the results will serve as personalized treatment based on the genetic profile, liquid biopsy and the microbiome. Genetic alterations and microbiome may be related to the differentiation of their immunogenicity and allow for further stratification of the relapse risk in patients with stages II and III CRC. Moreover, changes in the microbiome affect not only the immune response but also the systemic treatment response (chemotherapy, targeting, immunotherapy). 

The Cologramme aims to categorize the risk of relapse and provide guidance for treatment (or no treatment) decisions in daily clinical practice. Such an approach could (i). prevent unnecessary toxic treatment for patients with good prognosis (low risk of relapse), (ii). reduce the cost of managing patients with early disease while preserving system resources, and (iii). reduce the cost of dealing with side effects. Particularly nowadays, where new data are emerging to reduce the chemotherapy administration interval from 6 to 3 months in patients with stage III CRC, this approach becomes more valuable (24). In particular, the present study could highlight subpopulations of patients with a low risk of relapse and lead to the documentation of these results through a prospective clinical trial. Moreover, the determination of the treatment regimen may be associated with both the worsening on the quality of life of patients who will eventually be exposed to a stronger treatment than that needed and the significant economic costs resulting from the enhanced treatment. It could also have a significant impact on the design of more accurate and effective monitoring strategies and would contribute to further studies on the development of personalized treatment. That means an increased treatment efficacy, improved quality of life and overall patients’ survival. Ultimately, the proposed study is an important step towards medical precision, which takes into account personalized, precision medicine, personalized gene diversity, environment and lifestyle. 

## Figures and Tables

**Table 1:** An overview of studies associating particular genetic mutations to CRC recurrence.

	Mutation	Recurrence	Method of Detection	Sample Size	Study
LOW RISK	CDH10, COL6A3. SMAD4, TMEM132D, VCAN	mOS (80.4 m vs. 42.4 m) HR = 0.22; 95%CI (0.07–0.70); *p* = 0.0051	Exome sequencing and targeted capture sequencing	182	ASIAN COHORT Yu et al. (2014)
	AK2, CDC25A, HSPB1, BID, EIF4A2, ITGB1, MAP4K4, MMP12, RHOC, PTGES3, TERF2IP	HR = 1.812, 95% CI = 1.342–2.448, *p* < 0.001	Transcriptomic profiling using RNA-sequencing data	130	CIT COHORT Kim et al. (2019)
HIGH RISK	PIK3CA	Tumor recurrence *p* = 0.031 and poor OS (*p* = 0.044)	Sanger sequencing	228	Shen et al. (2016)
	APC	*p* = 0.023; 95% CI = 0.237–0.898	MassArray method	1227	Lan et al. (2021)
	BRAF	Multivariate analysis of OS 95%CI (1.398–6.186); *p* = 0.004	MassArray method	1227	Lan et al. (2021)
	NRAS	Multivariate analysis of OS 95%CI (0.827–3.044); *p* = 0.005	MassArray method	1227	Lan et al. (2021)
	KRAS	Recurrence after PM metastasectomy multivariate analysis *p* = 0.035 number of PMs (*p* = 0.037) lung as first site of recurrence after metastasectomy (*p* = 0.047)	Restriction fragment length analysis	44	Schweiger et al. (2013)
	KRAS in patients with synchronous CRLM	HR = 4.316 95%CI 1.973–9.845 *p* < 0.001	Polymerase chain reaction (PCR)-based primer extension assay	255	Sakai et al. (2021)
	Mkras, KRAS/NRAS/BRAF	3-year CSS (HR, 3.3; 95% CI, 1.6–6.5; *p* = 0.001	Sanger sequencing, next-generation sequencing (NGS), and/or by droplet digital polymerase chain reaction (PCR)	106	Brunsell et al. (2019)

mOS: median overall survival, HR: hazard ratio, CI: confidence interval, PM: pulmonary metastasectomy, CRLM: colorectal liver metastasis, CSS: cancer-specific survival.

**Table 2:** Studies on CTCs and ctDNA regarding resectable CRCs.

CTCs	CtDNA	Outcomes	Study
	ctDNA quantification	Earlier prediction and identification of recurrence	Bi et al. (2010)
Persistent post-operative CTCs in stage II/III colon cancer patients.		Strongly correlated with early relapse (*p* < 0.001; HR, 11.035; 95% CI: 4.396–32.190).	Lu et al. (2011)
CEACAM5mRNA-positive cells, in patients with resectable CRC.		Adverse prognostic factor associated with poor outcomes.	Vardakis et al. (2011)
CTCs in mesenteric circulation.		Indicators of recurrence.	Tseng et al. (2015)
CTCs vs. ctDNA.		ctDNA as a preferential specimen type for mutation screening in thoracic malignancies vs. CTC DNA.	Bi et al. (2015)
CTCs detection in stage I–III CRC patients after curative resection.		Significantly higher specificity, positive and negative predictive values, and accuracy for recurrence than CEA levels.	Chang et al. (2016)
		Inferior to CAF in recurrence prediction.	Rahbari et al. (2011)
Patients with ≥1 CTCs following stage I-III CRC resection.		Exercise was associated with a decrease in CTCs.	Brown et al. (2018)
Peripheral blood CEACAM5mRNA-positive CTCs, in patients with mCRC, especially in patients with KRAS and BRAF mutated tumors.		Adverse prognostic factor correlated with poor clinical outcome.	Messaritakis et al. (2018)
Post-operative—detection of CTCs.		Poor prognosis.	Yang et al. (2018)
	ctDNA-positive patients.	40-fold more likely to experience disease recurrence than ctDNA-negative patients (HR, 43.5; 95% CI, 9.8–193.5 *p* < 0.001).	Reinert et al. (2019)
	Post-surgical ctDNA status in stage III colon cancer.	Independently associated with RFI (HR, 7.5; 95% CI, 3.5–16.1; *p* < 0.001).	Tie et al. (2019)
	ctDNA vs. imaging and elevated tumor markers in early recurrence detection in patients with mCRC.	A useful tool for early detection of disease recurrence superior to imaging.	Benesova et al. (2019)
Pre-operative detection of ≥2 CTCs.		Recurrence/poor prognosis despite curative resection.	Arazzubi et al. (2019)
	ctDNA status in stage II–IV CRC who will undergo radical surgery.	*Ongoing.*	❖ GALAXY, Yukami et al. (2020)
	Observation vs. adjuvant CAPOX in high-risk stage II or low-risk stage III colon cancer, whose ctDNA status is negative post-operatively.	*Ongoing.*	VEGA, phase III (2020)
	Trifluridine/tipiracil vs. placebo in patients with positive ctDNA status post-resection.	*Ongoing.*	ALTAIR double-blind, phase III (2020)
	Post-resection ctDNA status and OS in CRC II or III.	*Ongoing.*	BESPOKE CRC prospective. (2020)
	Effectiveness of adjuvant chemotherapy based on ctDNA status in stage II CRC.	*Ongoing.*	MEDOCC-CrEATE, Schraa et al. (2020)
	ctDNA vs. CEA.	ctDNA showed higher sensitivity over CEA and consists of an independent predictive factor of recurrence.	Symonds et al. (2020)
	BRAF, NRAS and KRAS mutated ctDNA.	ctDNA following local treatment of mCRC is associated with an increased risk of recurrence and a short time to failure.	Boysen et al. (2020)
	KRAS mutated ctDNA.	Preoperative detection of KRAS mutated ctDNA was an independent factor related to both RFI (HR = 3.08; *p* = 0.012) and RFS (HR = 2.18; *p* = 0.044).	Nakamura et al. (2021)
High CTC score.		Poor prognosis/high recurrence risk in patients with stage III CRC but not in patients with stage II CRC.	Yang et al. (2021)

OS: overall survival, CEA: carcinoembryonic antigen, HR: hazard ratio, CI: confidence interval, CAF: circulating angiogenic factors, RFI: recurrence-free interval, RFS: recurrence-free survival, CRC: colorectal cancer, ctDNA: circulating tumor DNA, CTCs: circulating tumor cells. ❖ ctDNA results in the GALAXY trial, patients can be enrolled in either of the two distinct investigator-initiated phase III trials: the VEGA trial (treatment de-escalation) or the ALTAIR trial (treatment escalation). The VEGA trial assesses the non-inferiority of observation vs. adjuvant CAPOX in GALAXY participants who are high-risk stage II or low-risk stage III CRC and show absence of ctDNA one-month post-surgery. The ALTAIR trial evaluates the superiority of FTD/TPI over placebo in GALAXY participants with ctDNA status that remains positive after the standard therapy.

## References

[1] Vyslouzil K., Brychtova S., Zboril P., Skalicky P., Vomackova K., Bezdekova M., Brychta T. (2014). Unusual recurrent rectal carcinoma: A cancer field theory viewpoint. Biomed. Pap..

[2] Kunst N., Alarid-Escudero F., Aas E., Coupé V.M.H., Schrag D., Kuntz K.M. (2020). Estimating population-based recurrence rates of colorectal cancer over time in the United States. Cancer Epidemiol. Biomark. Prev..

[3] Duineveld L.A.M., van Asselt K.M., Bemelman W.A., Smits A.B., Tanis P.J., van Weert H.C.P.M., Wind J. (2016). Symptomatic and asymptomatic colon cancer recurrence: A multicenter cohort study. Ann. Fam. Med..

[4] Xu W., He Y., Wang Y., Li X., Young J., Ioannidis J.P.A., Dunlop M.G., Theodoratou E. (2020). Risk factors and risk prediction models for colorectal cancer metastasis and recurrence: An umbrella review of systematic reviews and meta-analyses of observational studies. BMC Med..

[5] Kim S.-E., Paik H.Y., Yoon H., Lee J.E., Kim N., Sung M.-K. (2015). Sex- and gender-specific disparities in colorectal cancer risk. World J. Gastroenterol..

[6] Arshad H.M.S., Tetangco E., Shah N., Kabir C., Raddawi H. (2016). Racial Disparities in Colorectal Carcinoma Incidence, Severity and Survival Times Over 10 Years: A Retrospective Single Center Study. J. Clin. Med. Res..

[7] Ohri A., Robinson A., Liu B., Bhuket T., Wong R. (2020). Updated Assessment of Colorectal Cancer Incidence in the U.S. by Age, Sex, and Race/Ethnicity. Dig. Dis. Sci..

[8] Lawler M., Alsina D., Adams R.A., Anderson A.S., Brown G., Fearnhead N.S., Fenwick S.W., Halloran S.P., Hochhauser D., Hull M.A. (2018). Critical research gaps and recommendations to inform research prioritisation for more effective prevention and improved outcomes in colorectal cancer. Gut.

[9] Genetics of Colorectal Cancer (PDQ®)–Health Professional Version-National Cancer Institute. https://www.cancer.gov/types/colorectal/hp/colorectal-genetics-pdq.

[10] Drew D.A., Devers T.J., O’Brien M.J., Horelik N.A., Levine J., Rosenberg D.W. (2014). HD chromoendoscopy coupled with DNA mass spectrometry profiling identifies somatic mutations in microdissected human proximal aberrant crypt foci. Mol. Cancer Res..

[11] Ebi H., Bando H., Taniguchi H., Sunakawa Y., Okugawa Y., Hatanaka Y., Hosoda W., Kumamoto K., Nakatani K., Yamazaki K. (2020). Japanese Society of Medical Oncology Clinical Guidelines: Molecular Testing for Colorectal Cancer Treatment, 4th edition. Cancer Sci..

[12] Afrǎsânie V.A., Marinca M.V., Alexa-Stratulat T., Gafton B., Pǎduraru M., Adavidoaiei A.M., Miron L., Rusu C. (2019). KRAS, NRAS, BRAF, HER2 and microsatellite instability in metastatic colorectal cancer-practical implications for the clinician. Radiol. Oncol..

[13] Yokota T. (2012). Are KRAS/BRAF Mutations Potent Prognostic and/or Predictive Biomarkers in Colorectal Cancers?. Anticancer. Agents Med. Chem..

[14] Bass A.J., Lawrence M.S., Brace L.E., Ramos A.H., Drier Y., Cibulskis K., Sougnez C., Voet D., Saksena G., Sivachenko A. (2011). Genomic sequencing of colorectal adenocarcinomas identifies a recurrent VTI1A-TCF7L2 fusion. Nat. Genet..

[15] Yu J., Wu W.K.K., Li X., He J., Li X.X., Ng S.S.M., Yu C., Gao Z., Yang J., Li M. (2015). Novel recurrently mutated genes and a prognostic mutation signature in colorectal cancer. Gut.

[16] Kim S.K., Kim S.Y., Kim C.W., Roh S.A., Ha Y.J., Lee J.L., Heo H., Cho D.H., Lee J.S., Kim Y.S. (2019). A prognostic index based on an eleven gene signature to predict systemic recurrences in colorectal cancer. Exp. Mol. Med..

[17] Shen Y., Han X., Wang J., Wang S., Yang H., Lu S.H., Shi Y. (2016). Prognostic impact of mutation profiling in patients with stage II and III colon cancer. Sci. Rep..

[18] De Sousa E Melo F., Colak S., Buikhuisen J., Koster J., Cameron K., De Jong J.H., Tuynman J.B., Prasetyanti P.R., Fessler E., Van Den Bergh S.P. (2011). Methylation of cancer-stem-cell-associated wnt target genes predicts poor prognosis in colorectal cancer patients. Cell Stem Cell.

[19] Domingo E., Church D.N., Sieber O., Ramamoorthy R., Yanagisawa Y., Johnstone E., Davidson B., Kerr D.J., Tomlinson I.P.M., Midgley R. (2013). Evaluation of PIK3CA mutation as a predictor of benefit from nonsteroidal anti-inflammatory drug therapy in colorectal cancer. J. Clin. Oncol..

[20] Lan Y.T., Chang S.C., Lin P.C., Lin C.C., Lin H.H., Huang S.C., Lin C.H., Liang W.Y., Chen W.S., Jiang J.K. (2021). Clinicopathological and molecular features of patients with early and late recurrence after curative surgery for colorectal cancer. Cancers.

[21] Vakiani E., Shah R.H., Berger M.F., Makohon-Moore A.P., Reiter J.G., Ostrovnaya I., Attiyeh M.A., Cercek A., Shia J., Iacobuzio-Donahue C.A. (2017). Local recurrences at the anastomotic area are clonally related to the primary tumor in sporadic colorectal carcinoma. Oncotarget.

[22] Schweiger T., Hegedüs B., Nikolowsky C., Hegedüs Z., Szirtes I., Mair R., Birner P., Döme B., Lang G., Klepetko W. (2014). EGFR, BRAF and KRAS status in patients undergoing pulmonary metastasectomy from primary colorectal carcinoma: A prospective follow-up study. Ann. Surg. Oncol..

[23] Sakai N., Furukawa K., Takayashiki T., Kuboki S., Takano S., Ohtsuka M. (2021). Differential effects of KRAS mutational status on long-term survival according to the timing of colorectal liver metastases. BMC Cancer.

[24] Brunsell T.H., Sveen A., Bjørnbeth B.A., Røsok B.I., Danielsen S.A., Brudvik K.W., Berg K.C.G., Johannessen B., Cengija V., Abildgaard A. (2020). High Concordance and Negative Prognostic Impact of RAS/BRAF/PIK3CA Mutations in Multiple Resected Colorectal Liver Metastases. Clin. Colorectal Cancer.

[25] Matteo B., Gaetano P., Delfina T., Riccardo M., Roberto S., Guglielmo P., Claudia C., Carmelo L., Carla C., Daris F. (2020). Immunohistochemical evaluation of microsatellite instability in resected colorectal liver metastases: A preliminary experience. Med. Oncol..

[26] Gmeiner W.H. (2021). Recent advances in our knowledge of mCRC tumor biology and genetics: A focus on targeted therapy development. Onco. Targets. Ther..

[27] Zhao Y., Wang C., Goel A. (2021). Role of gut microbiota in epigenetic regulation of colorectal Cancer. Biochim. Biophys. Acta Rev. Cancer.

[28] Liu W., Zhang X., Xu H., Li S., Lau H.C.-H., Chen Q., Zhang B., Zhao L., Chen H., Sung J.J.-Y. (2021). Microbial Community Heterogeneity Within Colorectal Neoplasia and its Correlation With Colorectal Carcinogenesis. Gastroenterology.

[29] Schulte am Esch J., Windmöller B.A., Hanewinkel J., Storm J., Förster C., Wilkens L., Krüger M., Kaltschmidt B., Kaltschmidt C. (2020). Isolation and Characterization of Two Novel Colorectal Cancer Cell Lines, Containing a Subpopulation with Potential Stem-Like Properties: Treatment Options by MYC/NMYC Inhibition. Cancers.

[30] Walcher L., Kistenmacher A.-K., Suo H., Kitte R., Dluczek S., Strauß A., Blaudszun A.-R., Yevsa T., Fricke S., Kossatz-Boehlert U. (2020). Cancer Stem Cells—Origins and Biomarkers: Perspectives for Targeted Personalized Therapies. Front. Immunol..

[31] Walker B.S., Zarour L.R., Wieghard N., Gallagher A.C., Swain J.R., Weinmann S., Lanciault C., Billingsley K., Tsikitis V.L., Wong M.H. (2020). Stem Cell Marker Expression in Early Stage Colorectal Cancer is Associated with Recurrent Intestinal Neoplasia. World J. Surg..

[32] Pisano A., Griñan-Lison C., Farace C., Fiorito G., Fenu G., Jiménez G., Scognamillo F., Peña-Martin J., Naccarati A., Pröll J. (2020). The Inhibitory Role of miR-486-5p on CSC Phenotype Has Diagnostic and Prognostic Potential in Colorectal Cancer. Cancers.

[33] Shreiner A.B., Kao J.Y., Young V.B. (2015). The gut microbiome in health and in disease. Curr. Opin. Gastroenterol..

[34] Wong S.H., Yu J. (2019). Gut microbiota in colorectal cancer: Mechanisms of action and clinical applications. Nat. Rev. Gastroenterol. Hepatol..

[35] Cheng Y., Ling Z., Li L. (2020). The Intestinal Microbiota and Colorectal Cancer. Front. Immunol..

[36] Eaden J.A., Abrams K.R., Mayberry J.F. (2001). The risk of colorectal cancer in ulcerative colitis: A meta-analysis. Gut.

[37] Heo G., Lee Y., Im E. (2021). Interplay between the gut microbiota and inflammatory mediators in the development of colorectal cancer. Cancers.

[38] Collins R.R.J., Patel K., Putnam W.C., Kapur P., Rakheja D. (2017). Oncometabolites: A new paradigm for oncology, metabolism, and the clinical laboratory. Clin. Chem..

[39] Gomes S.D., Oliveira C.S., Azevedo-Silva J., Casanova M.R., Barreto J., Pereira H., Chaves S.R., Rodrigues L.R., Casal M., Côrte-Real M. (2018). The Role of Diet Related Short-Chain Fatty Acids in Colorectal Cancer Metabolism and Survival: Prevention and Therapeutic Implications. Curr. Med. Chem..

[40] Ternes D., Karta J., Tsenkova M., Wilmes P., Haan S., Letellier E. (2020). Microbiome in Colorectal Cancer: How to Get from Meta-omics to Mechanism?. Trends Microbiol..

[41] Zackular J.P., Rogers M.A.M., Ruffin M.T., Schloss P.D. (2014). The human gut microbiome as a screening tool for colorectal cancer. Cancer Prev. Res..

[42] Lopez L.R., Bleich R.M., Arthur J.C. (2021). Microbiota Effects on Carcinogenesis: Initiation, Promotion, and Progression. Annu. Rev. Med..

[43] Dalmasso G., Cougnoux A., Delmas J., Darfeuille-Michaud A., Bonnet R. (2015). The bacterial genotoxin colibactin promotes colon tumor growth by modifying the tumor microenvironment. Gut Microbes.

[44] Rosendahl Huber A., Pleguezuelos-Manzano C., Puschhof J. (2021). A bacterial mutational footprint in colorectal cancer genomes. Br. J. Cancer.

[45] Iyadorai T., Mariappan V., Vellasamy K.M., Wanyiri J.W., Roslani A.C., Lee G.K., Sears C., Vadivelu J. (2020). Prevalence and association of pks+ Escherichia coli with colorectal cancer in patients at the University Malaya Medical Centre, Malaysia. PLoS ONE.

[46] Cheng W.T., Kantilal H.K., Davamani F. (2020). The mechanism of bacteroides fragilis toxin contributes to colon cancer formation. Malays. J. Med. Sci..

[47] Serna G., Ruiz-Pace F., Hernando J., Alonso L., Fasani R., Landolfi S., Comas R., Jimenez J., Elez E., Bullman S. (2020). Fusobacterium nucleatum persistence and risk of recurrence after preoperative treatment in locally advanced rectal cancer. Ann. Oncol..

[48] O’Dwyer P.J., Martin E.W. (1989). Viable intraluminal tumour cells and local regional tumour growth in experimental colon cancer. Ann. R. Coll. Surg. Engl..

[49] Yu S.Y., Xie Y.H., Qiu Y.W., Chen Y.X., Fang J.Y. (2019). Moderate alteration to gut microbiota brought by colorectal adenoma resection. J. Gastroenterol. Hepatol..

[50] Shogan B.D., Belogortseva N., Luong P.M., Zaborin A., Lax S., Bethel C., Ward M., Muldoon J.P., Singer M., An G. (2015). Collagen degradation and MMP9 activation by Enterococcus faecalis contribute to intestinal anastomotic leak. Sci. Transl. Med..

[51] Shogan B.D., Smith D.P., Christley S., Gilbert J.A., Zaborina O., Alverdy J.C. (2014). Intestinal anastomotic injury alters spatially defined microbiome composition and function. Microbiome.

[52] Olivas A.D., Shogan B.D., Valuckaite V., Zaborin A., Belogortseva N., Musch M., Meyer F.L., Trimble W., An G., Gilbert J. (2012). Intestinal Tissues Induce an SNP Mutation in Pseudomonas aeruginosa That Enhances Its Virulence: Possible Role in Anastomotic Leak. PLoS ONE.

[53] Colov E.P., Degett T.H., Raskov H., Gögenur I. (2020). The impact of the gut microbiota on prognosis after surgery for colorectal cancer–A systematic review and meta-analysis. APMIS.

[54] Chen Y., Peng Y., Yu J., Chen T., Wu Y., Shi L., Li Q., Wu J., Fu X. (2017). Invasive Fusobacterium nucleatum activates beta-catenin signaling in colorectal cancer via a TLR4/P-PAK1 cascade. Oncotarget.

[55] Rastogi Y.R., Saini A.K., Thakur V.K., Saini R.V. (2020). New insights into molecular links between microbiota and gastrointestinal cancers: A literature review. Int. J. Mol. Sci..

[56] Flemer B., Lynch D.B., Brown J.M.R., Jeffery I.B., Ryan F.J., Claesson M.J., O’Riordain M., Shanahan F., O’Toole P.W. (2017). Tumour-associated and non-tumour-associated microbiota in colorectal cancer. Gut.

[57] Yu T.C., Guo F., Yu Y., Sun T., Ma D., Han J., Qian Y., Kryczek I., Sun D., Nagarsheth N. (2017). Fusobacterium nucleatum Promotes Chemoresistance to Colorectal Cancer by Modulating Autophagy. Cell.

[58] Zhang S., Yang Y., Weng W., Guo B., Cai G., Ma Y., Cai S. (2019). Fusobacterium nucleatum promotes chemoresistance to 5-fluorouracil by upregulation of BIRC3 expression in colorectal caner. J. Exp. Clin. Cancer Res..

[59] Yu J., Feng Q., Wong S.H., Zhang D., Yi Liang Q., Qin Y., Tang L., Zhao H., Stenvang J., Li Y. (2017). Metagenomic analysis of faecal microbiome as a tool towards targeted non-invasive biomarkers for colorectal cancer. Gut.

[60] Gaines S., van Praagh J.B., Williamson A.J., Jacobson R.A., Hyoju S., Zaborin A., Mao J., Koo H.Y., Alpert L., Bissonnette M. (2020). Western Diet Promotes Intestinal Colonization by Collagenolytic Microbes and Promotes Tumor Formation After Colorectal Surgery. Gastroenterology.

[61] Gasser M., Lissner R., Nawalaniec K., Hsiao L.L., Waaga-Gasser A.M. (2020). KMP01D Demonstrates Beneficial Anti-inflammatory Effects on Immune Cells: An ex vivo Preclinical Study of Patients With Colorectal Cancer. Front. Immunol..

[62] Mantovani A., Allavena P., Sica A., Balkwill F. (2008). Cancer-related inflammation. Nature.

[63] Colorectal Cancer: Seed and Soil Hypothesis Revisited|Anticancer Research. https://ar.iiarjournals.org/content/34/5/2087.long.

[64] Huang Q.Y., Yao F., Zhou C.R., Huang X.Y., Wang Q., Long H., Wu Q.M. (2020). Role of gut microbiome in regulating the effectiveness of metformin in reducing colorectal cancer in type 2 diabetes. World J. Clin. Cases.

[65] Bryrup T., Thomsen C.W., Kern T., Allin K.H., Brandslund I., Jørgensen N.R., Vestergaard H., Hansen T., Hansen T.H., Pedersen O. (2019). Metformin-induced changes of the gut microbiota in healthy young men: Results of a non-blinded, one-armed intervention study. Diabetologia.

[66] Demb J., Yaseyyedi A., Liu L., Bustamante R., Earles A., Ghosh P., Gutkind J.S., Gawron A.J., Kaltenbach T.R., Martinez M.E. (2019). Metformin is Associated with Reduced Odds for Colorectal Cancer among Persons with Diabetes. Clin. Transl. Gastroenterol..

[67] Xi Y., Yuefen P., Wei W., Quan Q., Jing Z., Jiamin X., Shuwen H. (2019). Analysis of prognosis, genome, microbiome, and microbial metabolome in different sites of colorectal cancer. J. Transl. Med..

[68] Dejea C.M., Wick E.C., Hechenbleikner E.M., White J.R., Mark Welch J.L., Rossettid B.J., Peterson S.N., Snesrud E.C., Borisy G.G., Lazarev M. (2014). Microbiota organization is a distinct feature of proximal colorectal cancers. Proc. Natl. Acad. Sci. USA.

[69] DeDecker L., Coppedge B., Avelar-Barragan J., Karnes W., Whiteson K. (2021). Microbiome distinctions between the CRC carcinogenic pathways. Gut Microbes.

[70] Kim K., Castro E.J.T., Shim H., Advincula J.V.G., Kim Y.W. (2018). Differences regarding the molecular features and gut microbiota between right and left colon cancer. Ann. Coloproctol..

[71] Fong W., Li Q., Yu J. (2020). Gut microbiota modulation: A novel strategy for prevention and treatment of colorectal cancer. Oncogene.

[72] Pearson T., Caporaso J.G., Yellowhair M., Bokulich N.A., Padi M., Roe D.J., Wertheim B.C., Linhart M., Martinez J.A., Bilagody C. (2019). Effects of ursodeoxycholic acid on the gut microbiome and colorectal adenoma development. Cancer Med..

[73] Boursi B., Mamtani R., Haynes K., Yang Y.X. (2015). Recurrent antibiotic exposure may promote cancer formation-Another step in understanding the role of the human microbiota?. Eur. J. Cancer.

[74] Phipps O., Al-Hassi H.O., Quraishi M.N., Dickson E.A., Segal J., Steed H., Kumar A., Acheson A.G., Beggs A.D., Brookes M.J. (2021). Oral and intravenous iron therapy differentially alter the on-and off-tumor microbiota in anemic colorectal cancer patients. Cancers.

[75] Zhang X., Browman G., Siu W., Basen-Engquist K.M., Hanash S.M., Hoffman K.L., Okhuysen P.C., Scheet P., Petrosino J.F., Kopetz S. (2019). The BE GONE trial study protocol: A randomized crossover dietary intervention of dry beans targeting the gut microbiome of overweight and obese patients with a history of colorectal polyps or cancer. BMC Cancer.

[76] Food and Microbiome Longitudinal Investigation (FAMiLI). https://clinicaltrials.gov/ct2/show/NCT03293758.

[77] Gut Microbiome in Fecal Samples from Patients with Metastatic Cancer Undergoing Chemotherapy or Immunotherapy. https://clinicaltrials.gov/ct2/show/NCT02960282.

[78] Metagenomic Evaluation of the Gut Microbiome in Patients with Lynch Syndrome and Other Hereditary Colonic Polyposis Syndromes. https://clinicaltrials.gov/ct2/show/NCT02371135.

[79] Metabiomics Colon Cancer Clinical Research Study. https://clinicaltrials.gov/ct2/show/NCT02151123.

[80] Krebs M.G., Hou J.M., Ward T.H., Blackhall F.H., Dive C. (2010). Circulating tumour cells: Their utility in cancer management and predicting outcomes. Ther. Adv. Med. Oncol..

[81] Fiala C., Diamandis E.P. (2019). New approaches for detecting cancer with circulating cell-free DNA. BMC Med..

[82] Vietsch E.E., Wellstein A. (2018). Circulating DNA in cancer diagnosis and prognosis. Oncogenomics: From Basic Research to Precision Medicine.

[83] Konczalla L., Wöstemeier A., Kemper M., Karstens K.F., Izbicki J., Reeh M. (2020). Clinical significance of circulating tumor cells in gastrointestinal carcinomas. Diagnostics.

[84] Takeda K., Yamada T., Takahashi G., Iwai T., Ueda K., Kuriyama S., Koizumi M., Matsuda A., Shinji S., Ohta R. (2019). Analysis of colorectal cancer-related mutations by liquid biopsy: Utility of circulating cell-free DNA and circulating tumor cells. Cancer Sci..

[85] Bronkhorst A.J., Ungerer V., Holdenrieder S. (2019). The emerging role of cell-free DNA as a molecular marker for cancer management. Biomol. Detect. Quantif..

[86] Bi F., Wang Q., Dong Q., Wang Y., Zhang L., Zhang J. (2020). Circulating tumor DNA in colorectal cancer: Opportunities and challenges. Am. J. Transl. Res..

[87] Vidal J., Muinelo L., Dalmases A., Jones F., Edelstein D., Iglesias M., Orrillo M., Abalo A., Rodríguez C., Brozos E. (2017). Plasma ctDNA RAS mutation analysis for the diagnosis and treatment monitoring of metastatic colorectal cancer patients. Ann. Oncol..

[88] Reinert T., Schøler L.V., Thomsen R., Tobiasen H., Vang S., Nordentoft I., Lamy P., Kannerup A.S., Mortensen F.V., Stribolt K. (2016). Analysis of circulating tumour DNA to monitor disease burden following colorectal cancer surgery. Gut.

[89] Cohen J.D., Li L., Wang Y., Thoburn C., Afsari B., Danilova L., Douville C., Javed A.A., Wong F., Mattox A. (2018). Detection and localization of surgically resectable cancers with a multi-analyte blood test. Science.

[90] Wills B., Gorse E., Lee V. (2018). Role of liquid biopsies in colorectal cancer. Curr. Probl. Cancer.

[91] Bai Y., Zhao H. (2018). Liquid biopsy in tumors: Opportunities and challenges. Ann. Transl. Med..

[92] Sisson B.A., Uvalic J., Kelly K., Selvam P., Hesse A.N., Ananda G., Chandok H., Bergeron D., Holinka L., Reddi H.V. (2019). Technical and Regulatory Considerations for Taking Liquid Biopsy to the Clinic: Validation of the JAX PlasmaMonitor TM Assay. Biomark. Insights.

[93] Freidin M.B., Freydina D.V., Leung M., Fernandez A.M., Nicholson A.G., Lim E. (2015). Circulating tumor DNA outperforms circulating tumor cells for KRAS mutation detection in thoracic malignancies. Clin. Chem..

[94] Loaiza-Bonilla A., Benson A.B., Grothey A., Karimi M., Klempner S.J., Lin D., Mahtani R., Soares H.P. (2021). Use of Molecular Assays and Circulating Tumor DNA in Early-Stage Colorectal Cancer: A Roundtable Discussion of the Gastrointestinal Cancer Therapy Expert Group. Oncologist.

[95] Nakamura Y., Yokoyama S., Matsuda K., Tamura K., Mitani Y., Iwamoto H., Mizumoto Y., Murakami D., Kitahata Y., Yamaue H. (2021). Preoperative detection of KRAS mutated circulating tumor DNA is an independent risk factor for recurrence in colorectal cancer. Sci. Rep..

[96] Boysen A.K., Pallisgaard N., Andersen C.S.A., Spindler K.L.G. (2020). Circulating tumor DNA as a marker of minimal residual disease following local treatment of metastases from colorectal cancer. Acta Oncol..

[97] Symonds E.L., Pedersen S.K., Murray D., Byrne S.E., Roy A., Karapetis C., Hollington P., Rabbitt P., Jones F.S., LaPointe L. (2020). Circulating epigenetic biomarkers for detection of recurrent colorectal cancer. Cancer.

[98] Benešová L., Hálková T., Ptáčková R., Semyakina A., Menclová K., Pudil J., Ryska M., Levý M., Šimša J., Pazdírek F. (2019). Significance of postoperative follow-up of patients with metastatic colorectal cancer using circulating tumor DNA. World J. Gastroenterol..

[99] Tie J., Cohen J.D., Wang Y., Christie M., Simons K., Lee M., Wong R., Kosmider S., Ananda S., McKendrick J. (2019). Circulating tumor dna analyses as markers of recurrence risk and benefit of adjuvant therapy for stage III colon cancer. JAMA Oncol..

[100] Reinert T., Henriksen T.V., Christensen E., Sharma S., Salari R., Sethi H., Knudsen M., Nordentoft I., Wu H.T., Tin A.S. (2019). Analysis of Plasma Cell-Free DNA by Ultradeep Sequencing in Patients with Stages i to III Colorectal Cancer. JAMA Oncol..

[101] Yukami H., Saori M., Kotani D., Oki E., Taniguchi H., Nakamura Y., Kato T., Takemasa I., Yamanaka T., Shirasu H. (2020). 113TiP Prospective observational study monitoring circulating tumour DNA in resectable colorectal cancer patients undergoing radical surgery: GALAXY study in CIRCULATE-Japan. Ann. Oncol..

[102] Yukami H., Mishima S., Kotani D., Oki E., Taniguchi H., Nakamura Y., Kato T., Takemasa I., Yamanaka T., Shirasu H. (2020). P-120 Prospective observational study monitoring circulating tumor DNA in resectable colorectal cancer patients undergoing radical surgery: GALAXY study in CIRCULATE-Japan (trial in progress). Ann. Oncol..

[103] ｜NIPH Clinical Trials Search. https://rctportal.niph.go.jp/en/detail?trial_id=JapicCTI-205363.

[104] BESPOKE Study of ctDNA Guided Therapy in Colorectal Cancer. https://clinicaltrials.gov/ct2/show/NCT04264702.

[105] Schraa S.J., Van Rooijen K.L., Van Der Kruijssen D.E.W., Rubio Alarcón C., Phallen J., Sausen M., Simmons J., Coupé V.M.H., Van Grevenstein W.M.U., Elias S. (2020). Circulating tumor DNA guided adjuvant chemotherapy in stage II colon cancer (MEDOCC-CrEATE): Study protocol for a trial within a cohort study. BMC Cancer.

[106] Chakrabarti S., Xie H., Urrutia R., Mahipal A. (2020). The promise of circulating tumor DNA (ctDNA) in the management of early-stage colon cancer: A critical review. Cancers.

[107] Yang C., Wei C., Wang S., Han S., Shi D., Zhang C., Lin X., Dou R., Xiong B. (2019). Combined features based on preoperative controlling nutritional status score and circulating tumour cell status predict prognosis for colorectal cancer patients treated with curative resection. Int. J. Biol. Sci..

[108] Arrazubi V., Mata E., Antelo M.L., Tarifa A., Herrera J., Zazpe C., Teijeira L., Viudez A., Suárez J., Hernández I. (2019). Circulating Tumor Cells in Patients Undergoing Resection of Colorectal Cancer Liver Metastases. Clinical Utility for Long-Term Outcome: A Prospective Trial. Ann. Surg. Oncol..

[109] Yang C., Shi D., Wang S., Wei C., Zhang C., Xiong B. (2018). Prognostic value of pre-and post-operative circulating tumor cells detection in colorectal cancer patients treated with curative resection: A prospective cohort study based on iset device. Cancer Manag. Res..

[110] Chang Y.T., Huang M.Y., Yeh Y.S., Huang C.W., Tsai H.L., Cheng T.L., Wang J.Y. (2016). A prospective study of comparing multi-gene biomarker chip and serum carcinoembryonic antigen in the postoperative surveillance for patients with stage I-III colorectal cancer. PLoS ONE.

[111] Tseng J.Y., Yang C.Y., Yang S.H., Lin J.K., Lin C.H., Jiang J.K. (2015). Circulating CD133+/ESA+ cells in colorectal cancer patients. J. Surg. Res..

[112] Galizia G., Gemei M., Orditura M., Romano C., Zamboli A., Castellano P., Mabilia A., Auricchio A., de Vita F., Del Vecchio L. (2013). Postoperative Detection of Circulating Tumor Cells Predicts Tumor Recurrence in Colorectal Cancer Patients. J. Gastrointest. Surg..

[113] Rahbari N.N., Reissfelder C., Mühlbayer M., Weidmann K., Kahlert C., Büchler M.W., Weitz J., Koch M. (2011). Correlation of circulating angiogenic factors with circulating tumor cells and disease recurrence in patients undergoing curative resection for colorectal liver metastases. Ann. Surg. Oncol..

[114] Lu C.Y., Uen Y.H., Tsai H.L., Chuang S.C., Hou M.F., Wu D.C., Hank Juo S.H., Lin S.R., Wang J.Y. (2011). Molecular detection of persistent postoperative circulating tumour cells in stages II and III colon cancer patients via multiple blood sampling: Prognostic significance of detection for early relapse. Br. J. Cancer.

[115] Vardakis N., Messaritakis I., Papadaki C., Agoglossakis G., Sfakianaki M., Saridaki Z., Apostolaki S., Koutroubakis I., Perraki M., Hatzidaki D. (2011). Prognostic significance of the detection of peripheral blood CEACAM5mRNA-positive cells by real-time polymerase chain reaction in operable colorectal cancer. Clin. Cancer Res..

[116] Messaritakis I., Sfakianaki M., Papadaki C., Koulouridi A., Vardakis N., Koinis F., Hatzidaki D., Georgoulia N., Kladi A., Kotsakis A. (2018). Prognostic significance of CEACAM5mRNA-positive circulating tumor cells in patients with metastatic colorectal cancer. Cancer Chemother. Pharmacol..

[117] Nakamura Y., Yoshino T. (2018). Clinical Utility of Analyzing Circulating Tumor DNA in Patients with Metastatic Colorectal Cancer. Oncologist.

[118] Brown J.C., Rhim A.D., Manning S.L., Brennan L., Mansour A.I., Rustgi A.K., Damjanov N., Troxel A.B., Rickels M.R., Ky B. (2018). Effects of exercise on circulating tumor cells among patients with resected stage I-III colon cancer. PLoS ONE.

[119] GIC SG-GastroIntestinal Cancer Study Group. https://www.emkapes.gr/.

[120] West N.P., Hohenberger W., Weber K., Perrakis A., Finan P.J., Quirke P. (2010). Complete mesocolic excision with central vascular ligation produces an oncologically superior specimen compared with standard surgery for carcinoma of the colon. J. Clin. Oncol..

[121] Nagtegaal I.D., Van de Velde C.J.H., Van Der Worp E., Kapiteijn E., Quirke P., Van Krieken J.H.J.M. (2002). Macroscopic evaluation of rectal cancer resection specimen: Clinical significance of the pathologist in quality control. J. Clin. Oncol..

[122] Guinney J., Dienstmann R., Wang X., De Reyniès A., Schlicker A., Soneson C., Marisa L., Roepman P., Nyamundanda G., Angelino P. (2015). The consensus molecular subtypes of colorectal cancer. Nat. Med..

[123] Wood L.D., Parsons D.W., Jones S., Lin J., Sjöblom T., Leary R.J., Shen D., Boca S.M., Barber T., Ptak J. (2007). The genomic landscapes of human breast and colorectal cancers. Science.

[124] Vogelstein B., Kinzler K.W. (2004). Cancer genes and the pathways they control. Nat. Med..

[125] Sinicrope F.A., Sargent D.J. (2012). Molecular pathways: Microsatellite instability in colorectal cancer: Prognostic, predictive, and therapeutic implications. Clin. Cancer Res..

[126] Le D.T., Durham J.N., Smith K.N., Wang H., Bartlett B.R., Aulakh L.K., Lu S., Kemberling H., Wilt C., Luber B.S. (2017). Mismatch repair deficiency predicts response of solid tumors to PD-1 blockade. Science.

[127] Alves dos Santos K., Clemente dos Santos I.C., Santos Silva C., Gomes Ribeiro H., de Farias Domingos I., Nogueira Silbiger V. (2020). Circulating Exosomal miRNAs as Biomarkers for the Diagnosis and Prognosis of Colorectal Cancer. Int. J. Mol. Sci..

